# Recent Trends and Developments to Valorize Sheep and Goat Cheese Whey for Small and Medium-Size Enterprises

**DOI:** 10.3390/foods15071217

**Published:** 2026-04-03

**Authors:** Nayil Dinkçi, Vildan Akdeniz, Ayşe Sibel Akalın

**Affiliations:** 1 Department of Dairy Technology, Faculty of Agriculture, Ege University, Izmir 35100, Türkiye; nayil.dinkci@ege.edu.tr (N.D.); sibel.akalin@ege.edu.tr (A.S.A.); 2 Tire Vocational School, Ege University, Tire 35900, Izmir, Türkiye

**Keywords:** whey valorization, goat whey, sheep whey, small and medium-sized dairy companies, membrane processes, anaerobic digestion, biorefinery, whey fermentation, whey beverages, single-cell protein

## Abstract

Sheep and goat milk are mainly used for cheese manufacture in small livestock farms, giving rise to a large volume of whey. Sheep and goat cheese whey possess excellent and specific functional and nutritional characteristics. The valorization of these valuable by-products through physicochemical or biotechnological processes compatible with artisanal production are important in terms of sustainability, i.e., economic, social, and environmental impacts. The main challenges for whey processing in small and medium-size enterprises (SMEs) are the lack of equipment, construction and information as well as the small amounts of cheese whey generated from these plants. Membrane technology can be convenient to produce valuable by-products for small dairy plants in the presence of enough investment cost and whey amount. Biotechnological treatments covering anaerobic digestion systems and fermentation processes are advantageous for SMEs over physicochemical methods on investment cost. In these processes, efficient microorganisms are able to produce high-value natural products, biofuels, and biopolymers. Anaerobic digestion is a suitable method for goat and sheep cheese whey valorization in SMEs due to the small volumes. Additionally, bioconversion into fermented beverages is a good choice for cheese whey valorization in SMEs because of its low operational and equipment cost.

## 1. Introduction

Food processing industries generate a huge amount of food waste, depending on the increase in the food output, world population and industrial developments in food technology. Globally, the dairy industry is one of the most significant manufacturing sectors and dairy products constitute a primary proportion of food that is manufactured and consumed daily [1,2].

The development of innovative technologies used to increase the quality and productivity of dairy products, including cheese, caused the generation of an enormous amount of liquid waste. Cheese whey (CW), a greenish yellow liquid stream generating from the cheese curd during production, is the main by-product of the dairy industry [3].

The worldwide production of cheese whey is estimated to be nearly 200 million tonnes, with approximately 40 million tonnes being generated in the European Union [2,4]. About 50% of milk produced in Europe (148 million tonnes) was processed to cheese manufacture, releasing to 54.8 million tonnes of liquid cheese whey in 2020 [5]. The milk of dairy small ruminants, providing approximately 3.5% of world milk production, are mainly used for cheese production in SMEs and there is no certain information on their cheese and whey amount [6]. The global volume of this by-product is expected to rise to about 203–241 million metric tonnes by 2030, depending on the imputed yearly increase of 1–2% [7].

In the dairy industry, depending on its high organic and volumetric load, CW is considered and characterized by a severe hazard and an enormous opportunity for biochemical and bioenergy production. As the main polluting waste stream and by-product, cheese whey should be managed properly because of its high organic load, including high biochemical (35–60 gL^−1^) and chemical (50–102 gL^−1^) oxygen demands for the safety of humanity and the environment [8]. Although CW contains useful nutritional components as a by-product that can be processed into value-added products, it is generally treated as wastewater due to the large volumes manufactured and the lack of necessary equipment for its recovery and reuse [9]. Unfortunately, 50% of the cheese whey produced worldwide is still discharged into water sources without any treatment, which causes a serious environmental problem due to the dissolved oxygen depletion [2].

In fact, within dairy wastes, CW especially represents an important opportunity for the production of biochemical substances and value-added products such as bioactive peptides, bacteriocins, prebiotics, and enzymes, as well as bioenergy and functional foods, due to its high organic and inorganic nutritional content [8,10]. The composition of CW varies depending on the cheese processing method, the milk source (cow, sheep, goat, or buffalo), the feed type given to the animals, and the lactation period, as well as the composition and quantity of water (dairy wastewater) [10,11].

Two well-known types of whey are acidic and sweet wheys. Sweet whey is obtained from cheese curd after the enzymatic coagulation of casein proteins in milk by using chymosin. Acid whey is obtained from the activity of lactobacilli or by using lactic acid to coagulate casein in milk. It is also obtained from the acid curdled cheeses, such as cottage cheese [11,12]. Three different waste streams were obtained by cheese production containing CW, second cheese whey (SCW), and dairy wastewater that was obtained from the washing of dairy equipment. SCW is the by-product obtained from the whey cheese manufacture, retaining about 60% of the dry matter of original cheese whey [10].

Although a large part of cheese produced worldwide is manufactured from cow’s milk, there is a growing interest in goat and sheep milk products due to their nutritional and lower allergenic properties compared to cow milk. Moreover, these small ruminant milks are mainly utilized for the manufacture of special cheeses, which are assessed for their special flavor and texture. Depending on the increasing demand for goat and sheep cheeses, a global increase in cheese whey from these small ruminants can be seen clearly. Small and medium-size traditional cheese industries processing goat, sheep, and also bovine milk generally do not manage CW as a waste [12,13]. CW obtained from these plants is generally discarded to the environment directly or used for animal feed as the companies do not have facilities for whey and second cheese whey valorization. The challenges for the cheese whey treatment of small ruminants’ milk originate from the fact that small and medium-sized enterprises lack dimension to perform the required investments and technical qualification for valorization [12,14].

However, CW and SCW generated during the production of sheep and goat cheese have been demonstrated to contain highly valuable nutritional components for the functional food market. The functional dairy industry has developed new fermented milks or functional dairy beverages possessing nutritional and health effects with probiotics and/or prebiotics. On the other hand, the production of goat and sheep milk-based fermented dairy products increased due to consumer preferences for these products. Moreover, a great interest has appeared in minor components of goat and ovine cheese whey that play important functions in human health, such as non-digestible oligosaccharides and bioactive peptides [15,16]. On the other hand, anaerobic digestion of cheese whey for the biogas industry is a great opportunity for goat and sheep cheese manufacturers in small farms [17,18].

Within this context, cheese wastewaters of small ruminants’ milk have to be valorized to follow circular economic principles due to both their commercial value and their pollutant character for the environment. The present review focused on the recent studies on the proposed biotechnological strategies, applications, and possibilities in using and valorizing cheese whey of ovine and goat milks for small and medium-sized dairy plants.

## 2. Production and Properties of Sheep and Goat Cheese Wheys

The milk supply of the world is provided by a number of species, corresponding to 83% for cattle, 13% for buffaloes, 2% for goats and 1% for sheep [19]. A large number of sheep and goats are included in the dairy small ruminant sector, although their milk reflects only a minor part of world milk production [6]. Goat and sheep milk production is a growing industry that is an important section of the economy in several countries and essential to the well-being of millions of people worldwide [20]. The production facilities of most dairy goat and sheep systems are eco-friendly and play an important role in developing rural societies [6]. Dairy small ruminants, which account for about 21% of all ovine and goats globally, provide approximately 3.5% of world milk production. Despite the large numbers of these small ruminants (sheep and goat), the annual production of sheep and goat milk represented only 1.3% and 1.9%, respectively, of the global milk production in 2016. However, depending on the current production and increasing trend during the last 50 years, it is expected to increase by 26% for ovine milk and 53% for caprine milk by 2030 [6].

Dairy small ruminants are mostly located in subtropical-temperate regions of Asia, Europe and Africa. Dairy goats are intensified in low-income food shortage countries and also in technologically developed, high-income countries. Dairy sheep are located around the Mediterranean and Black Sea areas [6]. Mediterranean countries, especially, produce a quarter of the goat milk and also two thirds of ovine milk globally. Therefore, the farming of dairy small ruminants and the sustainability of their smallholdings play an important role in the economics of Mediterranean countries [21]. Globally, sheep milk (10.4 Mt) is mainly produced in Asia (45.6%), with a notable amount in Turkey and China, followed by Europe (29.0%) and Africa (24.5%). In 2018, 726,421 tonnes of ovine cheese were manufactured worldwide, 402,743 tonnes of which were produced in the EU28 alone [22,23]. World goat milk (15.3 Mt) is mostly produced in Asia (52.7%), with notable amounts in the Indian subcontinent and Africa (25.7%), Europe (16.6%) and followed by America (4.9%). Regarding goat cheese production in the world, Africa (44%) and Europe (38%) have the highest levels, followed by Asia (13.6%) [24].

A great variety of cheese processing methods are employed in the world from different species and even breeds of dairy animals. In Europe alone, 238 particular cheeses have a protected designation of origin or a certification of protected geographical indication [25]. On the other hand, dairy sheep and goat farming are gaining importance worldwide, and excellent cheeses are produced from sheep and goat milk. The specific taste and texture of these cheeses and also their nutritional (protein content) and health (low allergenicity) properties provide to humans a valuable alternative to cow cheese [13].

Sheep and goat milk are mainly processed into artisanal cheeses in traditional dairy plants at the farm level, and their by-product, whey, is not evaluated due to small volumes and lack of technical knowledge and investment cost [26]. In addition, as a significant part of cheese produced from goat and sheep milk is from SMEs, especially in developing countries, information on whey availability is highly inaccurate [27,28].

For SMEs, goat and sheep breeding in the family is a practical alternative in comparison to cattle livestock [29]. In 2021, the major part of the 31 million tonnes of milk that were produced from sheep and goats, to a large extent in developing and underdeveloped countries, is used for cheese manufacture [30]. The whey obtained from the cheese manufacturing is generally used for whey cheese production, resulting in a secondary whey that is used for animal feed or discharged [31].

Cow whey is rich in αlactalbumin, β-lactoglobulin, and glycomacropeptides, generally used in sports nutrition and producing functional foods. The average protein content of ovine milk is higher than in caprine or cow milk [15]. Especially, the high levels of fat, protein and calcium per casein unit in sheep’s milk lead to an excellent raw material for cheese manufacture. Sheep cheese whey is also a nutritional by-product for the dairy industry, as it has higher protein and mineral content compared to cow milk cheese whey [32,33]. Sheep whey is known for its high bioactive peptide content and significant amounts of lactoferrin, immunoglobulins, and oligosaccharides. Therefore, sheep whey is valuable in nutraceutical applications such as immune-enhancing supplements and the production of infant formulas and high-quality cheese [34].

Goat cheese whey is richer in nonprotein nitrogen compounds, free amino acids and nucleotides compared to bovine and sheep whey, making it advantageous for baby food production. It is rich in lysozyme, taurine, and casein phosphopeptides and has hypoallergenic and antimicrobial properties. Therefore, it is beneficial for medical nutrition, infant formulas and pharmaceutical applications. It is also richer in oligosaccharides and contains sialic acid, promoting the development of an infant’s brain. Innovations in membrane technologies, biorefinery integration, and fermentation for functional beverages can improve the sustainability and efficiency of goat and sheep whey processing [34].

The general composition of whey obtained during the production of cheese from bovine, ovine and caprine milk is given in Table 1 [12].

Ovine cheese whey that contains a higher level of whey proteins compared to goat and cow wheys is generally used for the manufacture of whey cheeses, through thermal precipitation of soluble proteins, leading to the release of SCW such as ricotta. However, this is not the best solution for the recovery of nutritional and economically valuable components of cheese whey nor for the elimination of the environmental problem, as SCW still contains almost all the lactose and 50% of the initial protein content [13]. For small and medium-sized dairy plants, the ultrafiltration process is suitable due to its acceptable costs and operation feasibility; on the other hand, biotechnological methods enable the production of high-value-added products with minimal adaptations to existing equipment and without the need for high capital costs, while pollution is reduced by all these methods [35].

## 3. Valorization of Sheep and Goat Cheese Whey for Small and Medium-Size Enterprises

Sheep and goat cheese whey should be valorized in terms of economic, social, and environmental aspects. However, the technologies used in valorization are not feasible for SMEs due to local and economic reasons [36,37].

Throughout history, cheese whey obtained from sheep, goat, bovine, or buffalo milks has been processed into dairy products such as whey cheeses, e.g., ricotta (Italy), requesón (Spain), requeijão (Portugal) and lor (Turkey), and other nutritional and medical products. Second cheese whey, such as scotta (Italy) and sorelho (Portugal), is the main by-product of whey cheese manufacture. In Italy, annually, 15% of cheese whey, that is generally produced from ovine milk, is used to manufacture ricotta cheese, causing around 1 million tonnes of SCW generation [12,38].

The production of artisanal and traditional sheep and goat cheeses is generally conducted in small–medium-size dairy plants. The whey of goat cheese or the second cheese whey of sheep milk is delivered to farms for feeding dairy animals and pigs or discharged into the environmental area or septic tanks for water purification plants [39].

In fact, cheese whey is recommended for use in organic fertilization of soil, both economically and environmentally. It has the potential to be used as a biological fertilizer as it contains essential minerals as well as proteins for plant growth. The addition of whey-based hydrogel improves water retention and increases the nutrient content in soil [40]. Abdelraouf et al. [41] reported that a 1.0% whey level could substitute for inorganic fertilizers for growing maize. Conte et al. [42] highlighted the importance of whey to improve soil quality and plant growth while decreasing confidence in chemical fertilizers.

SMEs have limited resources to valorize cheese whey in terms of equipment and technologies. Therefore, new valorization strategies should be simple and applicable. In this context, the low microbiological and physical stability of whey is one of the major challenges [37]. Regulatory and safety considerations are important in whey valorization due to raw whey’s high susceptibility to microbial contamination from cheese production by pathogens like *Listeria monocytogenes*, *E. coli* O157:H7, *Salmonella*, and *Staphylococcus aureus*, which produce heat-stable enterotoxins that can persist through spray drying and pose severe food safety risks in products like powders, concentrates, or beverages. Specialized treatment conditions—such as pasteurization (72 °C/15 s), bactofugation, and membrane filtration (UF/NF)—are essential not only to reduce microbial loads to standards (<10^5^ CFU/g aerobes, pathogen absence) but also to preserve whey components for utilization while complying with FDA/EFSA regs and preventing environmental pollution from high-BOD discharge [43]. dos Santos et al. [44] declared that thermal pasteurization at 75 °C for 5 min could provide the microbiological stability of goat cheese whey for at least 21 days and recommended the use of additional precautions, such as acidification or the use of bioprotective cultures and antimicrobials for an extended shelf life. In another study, the efficiency of direct acidification, nisin addition or *Lacticaseibacillus casei* inoculation was applied to pasteurized (75 °C/5 min) sheep and goat cheese wheys to extend their shelf life. Correspondingly, nisin or *L. casei* utilization was advised as a possible strategy to improve the valorization time of cheese wheys that can be used by small-scale enterprises or artisanal producers [37,45].

In order to extend the shelf life and provide the stabilization of heat, a treatment design was applied for sheep cheese whey [46], and nisin and lactic cultures were used in sheep and goat cheese whey [26]. For sheep cheese whey stabilization, a binomial temperature of 75 °C/6 min was determined as the best option, ensuring acceptable microbial load up to 14 days of storage and preserving its physicochemical properties [46]. The addition of nisin and/or *L. casei* to sheep and goat cheese whey extended the shelf life of both wheys up to 28 days, and the results were found to be suitable for application in whey preservation [26].

On the other hand, due to the limited resources of small- and medium-scale enterprises in cheese whey valorization, Guliyev et al. [36] recommended the optimization of whey treatment using magnetic nanoparticles to reduce the environmental problem of whey. The optimum parameters were determined to be pH 8.0 and 15 mL of MNPs and pH 7.74 and 15 mL of MNPs for N removal (%) and for COD and COD removal (%), respectively [36].

The main bottlenecks for the valorization of cheese whey produced by the SMEs are the relatively low amount of whey, difficulty in collecting whey between the geographically dispersed producers and problems in finding the initial investment cost. To overcome this difficulty, it is advised to form strategic partnerships between agricultural cooperatives, dairy companies, economic operators and research centers for the creation of novel systems for whey processing and the production of high-value outputs [47].

There are attractive studies to increase process efficiency of whey for SMEs by techno-economic analysis for different daily capacities. These whey recovery studies should also be modeled to identify the optimal plant size [48].

It is reported that an effective processing of whey requires an inflow of approximately 100–700 t/d, which is higher than the amount provided from an SME. Therefore, the SMEs could access large volumes of fresh whey, possessing guaranteed safety and quality specifications. In this regard, an effective traceability system for whey will provide a variety of data on location, and safety and quality of the products by means of automatic systems. In this field, the blockchain technology can be used for the quality control procedures as a data storage and useful tool for SMEs [48]. The application of blockchain technology has been designed for the protected designation of origin (PDO) cheese production [49], emerging economically to the biggest cooperative of dairy farmers in Turkey [50], the domestic dairy supply chain in Vietnam [51] and the local milk collection centers and small holder farmers in Kenya [52].

In the study of Banaszewska et al. [53], the added value of cheese whey valorization and the effect of integral valorization of cheese whey were evaluated on the profit of a dairy manufacturer. According to the modeling results, cheese whey valorization is very profitable and additional profit could be achieved by integral valorization.

Within this context, a great interest arises for the valorization of ovine and caprine cheese wheys and second cheese wheys produced by SMEs due to their high production volumes increasing day by day and valuable ingredients possessing lower allergenic and higher nutritional properties compared to cow cheese whey, associated with the environmental importance. Membrane technology can easily be implemented to valorize cheese whey in small dairy plants [12,54]. Biotechnological processes covering the use of anaerobic digestion systems and fermentation processes to produce microbial starters, single-cell proteins and oils, bioactive molecules, and biofuels from whey are important valorization techniques for SMEs [55,56]. In addition, the use of goat and sheep whey for beverage production could be an innovative solution for the food industry due to their nutritional and functional properties [7,54].

### 3.1. Integration of Membrane Processes

The techniques of separation and concentration of nutritional components are essential for the valorization of CW due to its high water content. Membrane technology can present a cost-effective and promising strategy to obtain nutritional and medicinal ingredients of sheep and goat whey by reducing its environmental impact. Membrane filtration processes are one of the most common separation techniques to recover valuable components of cheese whey. The integration of membrane processes to recover cheese whey compounds and to develop new products is very notable for the sustainability of these small- and medium-scale industries [13,39,57].

Membrane separation techniques, primarily UF and nanofiltration (NF), are frequently used in whey processing to separate whey components, especially proteins and lactose. By using ceramic and polymeric filtration membranes, high purity is provided in whey protein recovery with minimum use of chemicals, although the polymeric ones are evaluated to be more favorable [58].

Ultrafiltration (UF) is well-established in the dairy industry to obtain whey protein concentrates (WPCs) from cheese whey, providing the retention of the α-lactalbumin (α-La), β-lactoglobulin (β-Lg), immunoglobulin (Ig), serum albumin (SA) and other minor ones, such as lactoferrin (LF) and lactoperoxidase (LP). The other compounds with lower molecular weights or sizes, like bioactive peptides, lysozyme, or glycomacropeptide, can largely permeate through the membranes, together with lactose and minerals [57]. The dilution mode of UF retentates is applied to improve the permeation of smaller compounds and the separation of protein and lactose fractions [57]. In the later stages of UF, DF water is included, providing the removal of nonprotein substances and reducing the salt content of salty whey due to the high mineral and salt content of UF permeate that could restrict its use in nutrition directly [58]. Thereafter, the nanofiltration can be applied for the recovery of ultrafiltration permeates. In the same way, the use of dilution mode in nanofiltration can improve the separation of smaller solutes, providing a final permeate with a very low organic load. Macedo et al. [57] proposed an integrated membrane process for the recovery of goat cheese whey components for SMEs. The study covers filtration, centrifugation and pasteurization, followed by ultrafiltration and nanofiltration processes. Ultrafiltration was made by using membranes of 10 and 1 kDa. The fouling was negligible for both membranes. Membranes of 10 kDa provided higher permeate fluxes, lactose purity and better protein retention. It provided a favorable separation between protein and lactose fractions using ultrafiltration followed by diafiltration and then the recovery of almost all the lactose through the nanofiltration. The authors proposed the application of these integrated processes to small- and medium-scale goat cheese dairies to contribute to their sustainability. In addition, it was reported that this system is feasible and economically viable in small- and medium-scale cheese plants processing ca. 3500 L milk day^−1^ and even in smaller-scale dairies (i.e., processing ca. 500 L milk day^−1^) [39,57].

Nanofiltration can provide a number of advantages compared to other separation techniques, such as reverse osmosis, to recover valuable solutes from CW and to ensure cost–benefit on the lower pressure operations, depending on the specific characteristics of nanofiltration, including low energy consumption and high selectivity toward small solutes. Nanofiltration (NF) has generally been used to produce demineralized whey for the food and beverage industry [13]. In the study of Macedo et al. [13], the process performance was evaluated on the ultrafiltration permeates of goat and sheep whey to reduce their organic load and recover their lactose content. It was reported that both permeates had a much lower organic load, and the process performance was mostly affected by the osmotic pressure, that increases depending on the increasing lactose concentration. The authors proposed to apply lower transmembrane pressure to control osmotic pressure and decline permeate flux, as well as dilution to minimize mineral fouling.

Different protein concentrates based largely on bovine whey proteins are produced, mostly two different forms of whey protein, whey protein concentrate (WPC) containing between 35 and 80% protein and whey protein isolate (WPI) containing higher than 80% protein is produced. UF and/or DF are the most popular processes for the pre-concentration of whey in the manufacture of WPC and WPI, as well as other membrane techniques such as nanofiltration, reverse osmosis, microfiltration, and electrodialysis. As an effective membrane filtration technique, the combination of UF/DF is used in the production of WPI or WPC, and then the pasteurization and evaporation are applied to concentrated whey [12,39]. However, the fouling of membranes or the concentration polarization phenomena depending on cheese whey composition, membrane characteristics and process parameters can impair the permeability and separation characteristics of membranes. Whey proteins, minerals, and residues of processing, like lipids, enzymes, peptides, and microorganisms, are the major foulants of UF membranes. As regards membrane fouling arising from whey proteins, the membranes of softer, more hydrophilic, and neutrally charged ones have low fouling potential. The skimming process should be performed to prevent membrane fouling in cheese whey with a high lipid content. In ultrafiltration, the use of the pre-concentration and then the dilution mode to purify whey proteins improves the separation extent between retentate and permeate parts [39].

In the study of Li et al. [59] on ultrafiltration membranes, sulfonated polyacrylonitrile, methoxy-polyethylene glycol-block-polyacrylonitrile, and methoxy-polyethylene glycol-block-sulfonated polyacrylonitrile (mPEG-b-SPAN) were synthesized to improve the selectivity and antifouling properties of prepared membranes. The optimized M3 membrane, produced by blending methoxy-polyethylene glycol-block-sulfonated polyacrylonitrile with polyacrylonitrile exhibited superior fouling resistance, an irreversible contamination value and whey recovery, providing high retention rates on proteins and outstanding fouling resilience [59].

Macedo et al. [39] reported that an integrated process of ultrafiltration/nanofiltration is feasible for small–medium cheese producers. In that study, the ultrafiltration/diafiltration system was designed to recover protein fraction in the goat cheese whey and second cheese whey from sheep’s milk for a small–medium-scale dairy plant. A membrane system was installed depending on the experimental results of membrane tests, and for a daily production of approximately 3500 L of cheese whey. For the organic UF membranes, optimized operating conditions of transmembrane pressure (0.2 MPa) and feed circulation velocity (0.94 m s^−1^) were used. The average permeate flux of UF processes to attain to the desired volume concentration factor (VCF) of 4.0 varied from around 83 L h^−1^ m^−2^, at the beginning of the experiments, until about 54 L h^−1^ m^−2^. This provided a decrease in permeate flux of approximately 35%, increasing fouling resilience, according to the Cost–Benefit Analysis (CBA) and Sensitivity Analysis (SA) carried out to assess the profitability of that membrane installation to produce three valuable products from the liquid whey protein concentrates. This system was found to be economically viable in small–medium-sized cheese plants depending on the results of CBA and SA. Consequently, the installation of a common membrane unit to valorize the whey of small cheese dairies was proposed to obtain a high investment grant [39]. In addition, considering the feasibility of membrane filtration, Pires et al. [35] reported that small–medium-scale dairy plants can use the ultrafiltration process to increase the protein concentration of sheep’s and goat’s cheese whey for synbiotic kefir manufacture.

In small–medium-sized dairy plants, the production of CW and SCW protein concentrates derived from UF can be a solution for sustainability by using their by-products directly in functional food production. In addition, the combination of membranes and high-pressure processing could be applied for the valorization of caprine cheese wheys to improve α-La yield [60].

### 3.2. Biotechnological Processes

**Figure 1:**
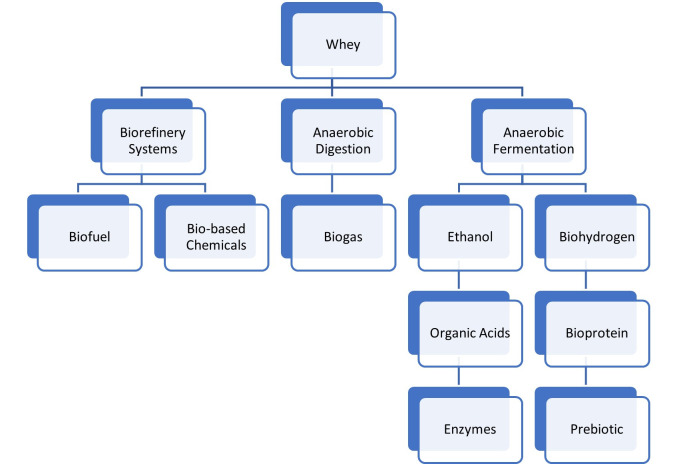
Methods for the biological valorization of whey.

Physicochemical treatments can be efficacious for dairy enterprises with elevated processing volumes and enough investment. Conversely, whey management poses difficulties for SMEs because they lack the necessary economic resources for proper treatment and valorization [61]. Biotechnological processes have recently become increasingly important for recovering valuable compounds from whey, and they are advantageous compared to physicochemical methods because microorganisms have versatile metabolisms and lead to the production of high-value-added products such as organic acids, bioproteins (single-cell proteins), enzymes, biopolymers and biofuels like biodiesel, ethanol, and biogas. These biotechnological processes include anaerobic digestion, fermentation, and biorefinery systems (Figure 1) [56,62,63]. Additionally, due to the high investment costs of physicochemical processes such as spray drying, the aforementioned bioprocesses are important options for the valorization of whey produced by small- and medium-scale cheese producers, particularly those producing sheep’s and goat’s cheese, which often lack large-scale treatment infrastructure [64]. Small and medium-sized enterprises can feasibly and affordably integrate whey valorization processes such as bioethanol production, single-cell protein (SCP) production and biorefinery systems using biotechnological processes. This allows them to leverage existing dairy waste without making major capital investments with simple adaptations to existing operating equipment. Simple adaptations include batch fermenters and ultrafiltration for lactose concentration, as well as forming partnerships to scale up production and generate revenue from by-products, all while reducing pollution [65].

In addition, biotechnological whey valorization usually reduces net environmental burdens compared with direct disposal. Using whey as a feedstock for bioproducts, such as bioproteins, lactic acid, bioplastics, or biogas, can lower carbon intensity. At the same time, converting whey rather than discharging it markedly cuts eutrophication potential by preventing high organic, nitrogen, and phosphorus loads from entering water bodies. It is stated that anaerobic digestion is the most sustainable option for the lowest environmental impact, reducing greenhouse gas and eutrophication, and producing biogas. However, it requires centralized facilities to minimize transport emissions. The production of ethanol, lactic acid, etc., through fermentation reduces waste but increases water/energy use. On the other hand, whey powder production has high carbon intensity from energy-intensive spray drying, while the production of WPC/WPI lowers the overall dairy carbon footprint by substituting milk powder. Different whey valorization pathways show complex environmental effects. But compared to direct disposal of whey, which causes severe eutrophication (0.23–0.26 kg N/P equivalents per ton whey due to high-BOD/COD discharge), all valorization pathways have positive environmental effects [65,66].

#### 3.2.1. Anaerobic Digestion

Anaerobic digestion is an effective biochemical process and a well-established, environmentally friendly technology for generating energy from whey. It involves the degradation of organic matter in whey without oxygen through a series of biochemical reactions. This process produces various substances, including biofuels such as hydrogen, methane and ethanol, as well as bio-based products such as carboxylic acids, proteins, and biopolymers [64].

Anaerobic digestion is the most feasible and sustainable solution for small- and medium-scale businesses, particularly those dealing with sheep and goat cheese, due to the small volumes and compositional differences in the whey involved. This process reduces environmental impact and enables energy recovery where high-tech alternatives are not economically viable. It should therefore be a fundamental component of waste management strategies, particularly for small- and medium-scale traditional cheese producers, especially in developing countries [1].

Anaerobic digestion of whey produces methane or biogas. This process involves three steps: lactose (and protein) hydrolysis, fermentation (acidogenesis, acetogenesis), and methanogenesis. The final step produces methane, which can be used for energy, as fuel or electricity. The organic matter in whey is oxidized under anaerobic conditions in a microbial fuel cell to produce electricity [61,63]. In this context, anaerobic digestion is a feasible option for SMEs. It leads to a decrease in chemical oxygen demand, the production of biogas, and the conversion of whey into a by-product that is rich in nutrients and stable, which can be used as fertilizer. Biogas could be used to heat milk boilers instead of diesel or gas, reducing energy bills and greenhouse gas emissions [64].

Biogas production from whey has high potential for methane via anaerobic digestion due to its organic richness. It is feasible through mono-digestion under optimized anaerobic digestion conditions such as mesophilic temperatures (30–40 °C), pH stabilization around 6.5–7.5, balanced nutrient profiles, optimal C/N ratios (20 to 30), and pretreatment to enhance biodegradability [67,68]. However, mono-digestion causes instabilities, because the high organic load and low alkalinity of whey can cause acidification due to the production of volatile fatty acids during anaerobic digestion, affecting methanogenic activity and process stability, and consequently reducing biomethane yield [69].

Thus, the co-digestion of whey with appropriate substrates is recommended for biogas production when mono-digestion risks instability from rapid acidification, nutrient imbalances (e.g., low or excessive C/N ratio), or a high overload of volatile solids [3,68]. Suitable materials for anaerobic digestion include agricultural residues, agro-industrial by-products, animal manure, organic urban waste, livestock residues and wastewater, with low capital expenditure [55]. de Albuquerque et al. [27] studied the co-digestion of whey with glycerin at a thermophilic temperature of 55 °C as a potential solution for small- to medium-sized dairy industries. They found that the highest methane productivity (203 molCH_4_ m^−3^ day^−1^) was achieved when the reactor was operated in fed-batch mode at 55 °C with an influent composition of 25% cheese whey and 75% glycerine on a chemical oxygen demand basis, with 68% of all influent chemical oxygen demand being removed. Comino et al. [70] and Hublin and Zelić [71] respectively found that the co-digestion 50% whey and 50% cow manure at 35 °C, and the co-digestion of whey and cow manure at 55 °C with an optimum mixing ratio of 10:90, increased the generated methane yield. Furthermore, Rincón-Pérez et al. [72] co-digested cheese whey with hydrolyzed microalgal biomass, achieving an increased methane production.

In addition, anaerobic digestion of whey offers small and mid-size dairy industry revenue from biogas energy and waste cost savings. Mainardis et al. [68] indicated that small Italian dairies (processing 3000–10,000 L milk/day) can cover 70–90% of electric (0.009–0.133 kWh/kg milk) and thermal (0.247–0.557 MJ/kg milk) needs via biogas from whey, yielding methane up to 437 NmL CH_4_/g VS and cutting energy costs by €0.0079–0.0308/kg milk.

In conclusion, although there are limitations to anaerobic digestion, such as the low pH and high lactose content of whey (which results in the need for pre-treatments), the production of a relatively low-value product (biogas) and instability in small digesters, it has advantages, such as the production of biogas for on-site energy needs (covering 50–100% of SME requirements), a reduction in pollution load and low cost for SMEs in developing regions [68,73].

Furthermore, anaerobic digestion of whey can be achieved through single-stage and two-stage processes. Employing a two-stage process facilitates the optimization of operating conditions for hydrolysis, acidogenesis, and methanogenesis, thus enabling the recovery of hydrogen produced during the first fermentation phase. Anaerobic digestion can therefore be used as part of an integrated treatment process in various biorefineries [55].

#### 3.2.2. Fermentation

Fermentation can be a sustainable approach for whey management and valorization. Since whey is a good nutrient source for microorganisms, commercially valuable products such as ethanol, single-cell proteins, organic acids, biogas, enzymes, probiotics, bacteriocins, exopolysaccharides, and bioplastics can be produced through microbial fermentation (Table 2) [63,74,75]. Another advantage of fermentation is the conduction of the processes at temperatures around 25–30 °C, providing energy saving [76]. In addition, it can be an alternative to anaerobic digestion, since methanogenic activity is affected because of whey’s high organic matter content with inadequate bicarbonate alkalinity [69].

Whey, a biodegradable waste, offers a valuable opportunity for the production of green energy and biochemistry. Among the numerous value-added products that can be obtained from whey fermentation, ethanol is the most prominent due to its status as the most widely used alternative biofuel worldwide [91]. From a renewable energy standpoint, whey has benefits over other food-related fermentation feedstocks, like corn, because it is a by-product. The use of whey ethanol is also suitable for the production of food and beverages because of its edibility [92].

##### Ethanol

Various yeast species, such as *Saccharomyces cerevisiae*, *Candida pseudotropicalis*, and *Kluyveromyces marxianus* (formerly *Kluyveromyces fragilis*), are commonly used for bioethanol production from whey through fermentation [93]. However, due to its high utilization value of lactose, *K. marxianus* has been the most commercially preferred microorganism. At least 95% of the lactose of unconcentrated whey is utilized by *K. marxianus* with a conversion efficiency of 80–85%, indicating the theoretical value of 0.538 kg ethanol/kg lactose consumed [45]. The low lactose content in whey is a key challenge in producing bioethanol from whey. To get around this problem, methods like ultrafiltration and reverse osmosis can be used to increase the amount of lactose in whey, which leads to a higher amount of bioethanol. Another option to increase the sugar concentration in whey is to mix it with condensed materials that are high in sugar, such as molasses [94]. Furthermore, using *Kluyveromyces marxianus* strains, which are capable of metabolizing lactose, has shown encouraging results in increasing bioethanol production from whey. Besides *Kluyveromyces* strains, *S. cerevisiae* was also studied for lactose fermentation from whey. Initially, *S. cerevisiae* was used in the fermentation of pre-hydrolyzed lactose solutions as mixtures of glucose and galactose. Then, *Saccharomyces cerevisiae* strains capable of efficiently utilizing lactose have been created by advanced genetic manipulation techniques. Thus, ethanol productivity was improved [95].

Temperature and pH are also important factors which affect ethanol productivity. Tesfaw et al. [78] found that significant amounts of ethanol were obtained from both pH-uncontrolled (3.9) and pH-controlled (5.0) whey. He also noted that the pH-controlled whey had a higher ethanol yield. Furthermore, he stated that high amounts of ethanol could be produced using *K. marxianus* yeast at temperatures up to 45 °C. Tesfaw et al. [78] also studied some supplements, such as peptone, yeast extract, ammonium sulfate, and urea, in whey to investigate the requirement of additional nutrients for ethanol optimization. Accordingly, the ammonium sulfate and peptone enhanced ethanol productivity, but yeast extract and urea depressed the yeast ethanol fermentation capability.

In conclusion, despite limitations, such as a slow start-up (20–40 days), sensitivity to inhibitors such as high lactate levels in acid whey, and the risk of methane leakage, fermentation yields high-value products from whey, such as ethanol, single-cell protein, organic acids, etc. It also improves whey stability and nutrition, and can be integrated with dairy processes. Compared to spray drying, it avoids thermal damage to proteins. It is one of the important and feasible methods in whey valorization for SMEs [73,95]. Currently, SMEs are unable to produce value-added products from their waste due to the cost of the necessary technology. Some cheese factories only process around half of their whey into powder or condensed whey, rather than producing ethanol, single-cell proteins, and organic acids. The remaining whey is discharged into the drainage system. Limited studies demonstrate the economic feasibility of producing ethanol from whey. A study by Utama et al. [96] on joint cost allocation for converting cheese-making waste (whey) into ethanol and organic liquid fertilizer using the market price method demonstrated the strong economic feasibility of small-scale operations. In this study, bioethanol production was found financially beneficial, reducing the cost of the main product by 14.73%, saving on disposal costs, and reaching the break-even point in under 4 months. Full production reached the break-even point rapidly via ethanol at ~US$3/L. The financial outlay for the study amounted to US$17,197.28, with 24,000 liters of whey undergoing treatment on a monthly basis. Low-cost, semi-anaerobic plastic containers are suitable for SMEs, as they turn waste into profit while reducing pollution and disposal costs. In addition, using distillery waste for biofertilizer production increased the economic viability of ethanol production [65,96].

##### Bioprotein/Single-Cell Protein

Moreover, it is feasible to biotransform whey into food-grade bioprotein/single-cell protein through fermentation [63]. Whey, particularly lactose, is used as a substrate for single-cell protein (SCP) production since it is a carbon source, in the meantime reducing whey pollution load. SCP can be produced from bacteria, fungi, and algae-based valorization of whey. Among the various microorganisms, yeasts, including *Kluveromyces*, *Candida*, and *Saccharomyces* spp., are the most commonly used microorganisms for the biotransformation of whey into SCP, as they can ferment lactose and proliferate in equilibrium in the whey/whey permeate. Yeast-derived SCP possesses elevated protein content and also provides B-complex vitamins and beta glucans-derived fiber [8,11]. However, there are some difficulties during this bioconversion process including contamination during fermentation, low COD removal efficiency, final product quality (amino acid profile of SCP), on-site whey processing in small- and medium-scale industries, and low economic profit [97]. To reduce the risk of contamination; high temperature and low pH can be selected as process conditions, and a suitable bacteriological quality can be obtained by pasteurization of the whey permeate at 80 °C [92]. A mixed culture, which could grow on the metabolites of lactose utilizing yeast, can be used to improve the product quality and increase COD removal. In addition, continuous fermentation can be used to increase economic profits by reducing process costs and enabling large volumes of whey to be bioconverted. It can also contribute to increasing COD removal [97].

##### Organic Acids

It is also possible to produce lactic acid by the fermentation of whey, as it is an ideal growth medium for various lactic acid bacteria, leading to yields of 70–90% lactic acid. For instance, poly(vinyl alcohol)-immobilized *Lactobacillus plantarum* has been used to ferment whey and other renewable feedstocks, achieving high lactic acid productivity in a stable, reusable system that enhances efficiency for industrial scales [84]. Similarly, non-pathogenic *Lactobacillus acidophilus* treatment of cheese whey not only generates biohydrogen but also co-produces lactic acid alongside other organics, demonstrating whey’s versatility as a substrate while addressing waste valorization. However, the fermentation of whey to produce lactic acid has its drawbacks, particularly the high cost of recovery, which remains a key challenge despite microbial optimizations [83].

Recent studies have expanded organic acid production from whey to include succinic, propionic, citric, and acetic acids, leveraging diverse microorganisms. *Actinobacillus succinogenes*, for example, efficiently converts whey and lactose into succinic acid via batch fermentation, yielding up to 54.5 g/L with high selectivity, highlighting whey’s potential as a low-cost carbon source for this valuable platform chemical used in bioplastics and pharmaceuticals [85]. *Kluyveromyces marxianus* further broadens this scope through the bioconversion of electro-activated whey and permeate, simultaneously producing lactic acid, acetic acid, propionic acid, and citric acid precursors, along with ethanol and single-cell protein, in an integrated process that maximizes resource recovery [76].

These advancements underscore whey’s role in sustainable biosystems, where tailored fermentation conditions and microbial strains mitigate recovery costs and boost yields of high-value acids for food, pharma, and green chemistry applications.

##### Prebiotics

In addition, whey lactose can be used as a substrate for the production of prebiotics, such as galactooligosaccharides (GOSs), lactulose, and lactobionic acid through enzymatic, microbial, fungal or microalgal processes. Some of these compounds can also be used as food additives [91]. GOS is the most notable of these prebiotics, and it can be synthesized from lactose using several methods: acid hydrolysis (the chemical route), glycosyltransferase, and transglycosylation. Acid hydrolysis, which has low product specificity and produces undesirable mixtures of disaccharides and trisaccharides, is non-applicable for the food industry [9]. The use of glycosyltransferases, which need activated nucleotide sugar donors, in GOS synthesis have limited industrial application. Because this process requires a multi-enzyme cascade, it is not economically viable [3,9]. The transglycosylation activity is the most common and frequently used method in the industrial production of GOS due to proper reaction conditions, low operating cost, and no need for purified substrates. In this method, the use of β-galactosidase enzyme, which is relatively cheap and does not cause the formation of undesirable by-products, is the most preferred [9]. *Lactobacillus delbrueckii* subsp. *bulgaricus* and *Streptococcus thermophilus* strains, and also yeast strains such as *Kluyveromyces marxianus* and *Candida pseudotropicalis*, can be used for their high β-galactosidase activity to synthesize GOS using whey as the substrate [89]. The β-galactosidase enzyme catalyzes a double reaction. Firstly, lactose is hydrolyzed into glucose and galactose by the enzyme, and galactose–enzyme intermediate is formed. This galactose–enzyme intermediate is then transferred to a glycosyl receptor (lactose) via transglycosylation, forming GOS [9].

##### Others

Whey proteins are also transformed into bioactive peptides through enzymatic or fermentation processes. Whey supports the growth of bacteria, mushrooms, and microalgae, acting as a substrate, thus providing the generation of high-value compounds such as polysaccharides, carotenoids, and chlorophyll [90]. Certain strains exhibit accelerated growth when cultivated on whey as a substrate, yielding elevated antioxidant activity, primarily due to whey proteolysis. Consequently, whey fermentation offers considerable promise for waste valorization, thereby facilitating the production of bioactive compounds [4].

Studies have shown that various strains of bacteria, yeast, algae and fungi can grow on whey to produce enzymes, including proteases, amylases, polygalacturonases and β-galactosidases [87,98]. However, β-galactosidase production is the most prominent, and the most research has been conducted on this topic. The microorganisms most commonly studied and considered most suitable for producing β-galactosidase from whey are *Kluyveromyces marxianus*, *Kluyveromyces lactis*, and *Saccharomyces fragilis* [74].

Depending on the type of microorganism, biogas and bioplastics can also be obtained as a result of the fermentation of cheese whey [99]. The dark fermentation process is an important biological method for the production of biohydrogen from cheese whey [62]. It is carried out by hydrogen-producing microorganisms, such as facultative anaerobes like *Enterobacter* spp. and obligate anaerobes like *Clostridium* spp. During this process, microorganisms grow on organic materials through oxidation, increasing their biomass and energy. Only one third of the energy stored in biomass is used to make biohydrogen; the rest is converted into liquid waste containing different metabolites. These metabolites can be used as raw materials for producing bioplastics, biobutanol, methane, and phosphate-solubilizing biofertilizers. The production of different metabolites during hydrogen production can be extended to the hydrogen refinery approach, as several other metabolites are also being produced simultaneously [62]. There has recently been a renewed focus on utilizing whey in the context of developing biorefineries [91].

### 3.3. Production of Whey-Based Beverages

Various technological solutions to increase the production of whey-based foods have been studied by researchers. From an economic and technological perspective, the production of whey-based beverages offers one of the most viable options to SMEs because they turn a waste stream into a low-investment, high-value product without needing a full-scale biorefinery or complex whey processing systems. This process is also familiar to current dairy experts [93]. Ultrafiltration on an SME scale, combined with a simple formulation involving flavoring, pasteurization, bottling, etc., can convert liquid whey concentrates directly into ready-to-drink beverages or fermented drinks using existing dairy equipment. Probiotic drinks, fruit-based fermented beverages, or kefir-like products have the potential to create high added value with low investment from whey. Thus, a product considered waste can be processed at low cost and transformed into a new sales item. This would not only solve the waste management problem for SMEs but also provide an opportunity for additional profit [100].

Whey-based drinks are highly nutritious and protein-rich, and can be adapted to meet various functional requirements (e.g., sports, children, health-conscious consumers), helping SMEs to differentiate their products and capture premium market segments with relatively low R&D and marketing costs. Using whey as a base reduces ingredient costs compared with reconstituted milk-based drinks, while also cutting disposal treatment expenses and improving margins, particularly for SMEs that would otherwise treat whey as effluent [101]. The key point in developing whey-based beverages lies in considering the nutritional and functional properties of the whey used, as well as meeting the expectations of conscious consumers who demand innovative, enhanced-functionality products. Various whey-based beverages on the market are presented as value-added and functional foods, and this number is steadily increasing [45]. Whey-based beverages can be produced as non-fermented and fermented whey-based beverages [100].

When developing a refreshing whey beverage, it is crucial to combine whey with fruit bases such as fruit juice and pulp in order to suppress negative aspects such as sediment formation, a salty or sour taste, and the odor of cooked milk, and to achieve an acceptable sensory quality. Non-fermented whey-based beverages are produced by mixing sweet or acidic whey with fruit juice such as orange, blackcurrant, tropical fruits, berries, black mulberry, prickly pear juice, and often with the addition of CO_2_. Furthermore, fruit powders, herbs, spices, flavoring compounds, and stabilizers can be added to whey beverages to enhance sensory quality and improve functionality [102,103]. Although a non-fermented, functional, whey-based beverage is the most practical way to valorize whey, the dry matter in the fruit can sometimes cause precipitation. Therefore, deproteinized whey or whey permeate, which is left over after ultrafiltration, has recently become a more popular way of avoiding undesirable sediment formation and clouding. Additionally, fermentation is another possible option [45].

The elevated nutritional value of whey renders it a commendable raw, sustainable material for the conception of new health-promoting foods. Fermented beverages have been acknowledged by conscious consumers worldwide for their therapeutic properties [104]. Whey can be transformed into a high-value product through a relatively simple, low-cost fermentation process using yogurt cultures and/or probiotic bacteria. These beverages can be easily produced by SMEs using their existing yogurt or fermented milk production equipment [100]. In whey fermentation, starter cultures and/or probiotic cultures that can metabolize lactose are commonly used, such as *Lactobacillus (rhamnosus*, *plantarum*, *casei*, *acidophilus*, *gasseri*, *reuteri*, *paracasei*, *debrueckii* subsp. *bulgaricus*), *Bifidobacterium (breve*, *bifidum)*, *Streptococcus thermophilus*, *Propionibacterium freudenreichii* subsp. *shermanii* and *Kluyveromyces marxianus* [104]. The most common whey drinks consist of a mixture of whey and fruit juice. Rivella, which has been produced and sold in Switzerland since the early 1950s from carbonated whey permeate flavored with extracts of various herbs, is one of the oldest examples of this [95]. Table 3 presents recent studies on the development of fermented and non-fermented whey-based beverages. As seen from the table, it is possible to valorize whey into beneficial and high-value-added beverages, as well as reducing whey waste. Combinations of whey and fruit juice/pulp, whether fermented or non-fermented, are increasingly popular in the functional beverage market. This is because they combine the health benefits of whey with the positive effects of vitamin C, β-carotene, mineral salts, dietary fibers and phenolic compounds found in fruits. Fermented fruit-whey beverages may also promote probiotic delivery [45].

Whey is also used in the production of alcoholic dirnks such as low alcohol drinks (<1% alcohol), whey beer, whey wine, and whey champagne [95].

In the case of manufacturers producing sheep and goat cheese, the nutritional value of whey makes it especially valuable. Goat dairy products have the potential to be used in the production of numerous functional foods. This is because they are excellent carriers of probiotics and offer a range of health benefits, including improved digestibility, a higher buffer capacity, lower cholesterol content and a higher calcium content than cow’s milk. Fermentation with starter cultures and/or probiotic cultures better reveals the potential of goat dairy products to be used as functional foods [105]. In this context, SMEs can easily apply whey-based beverage valorization because they utilize existing whey flows, use simple, scalable processing methods, and open up niche, high-value markets without requiring substantial initial investment. SMEs can develop their own unique fermented whey beverages, enriched with fruits or different flavors, tailored to their production processes, thereby increasing their competitiveness and contributing to a sustainable production model.

### 3.4. Biorefinery Systems

The potential of waste and its importance for sustainability is now widely recognized, and this has led to the introduction of biorefinery systems for the production of biofuels and various bio-based chemicals [10]. In this framework, whey, the main waste/by-product of the dairy industry, plays a significant role due to its potential use in biorefineries. The International Energy Agency defines biorefining as the sustainable processing of biomass into marketable products and energy [91]. There are three types of biorefinery: first-, second- and third-generation. First-generation biomass comes from food or derived crops, e.g., sugar cane or corn. These biomasses are optimal for producing biofuels, but are associated with socio-economic and environmental issues [112]. Second- and third-generation biorefineries use lignocellulosic and algae biomass, respectively. These overcome the main disadvantage of first-generation feedstocks, i.e., a stable price and no need for extra land. However, the lower carbon content of second- and third-generation biomasses limits biofuel production, particularly for bioethanol. Cheese whey, which is generated in large amounts and has a high carbon content, is being used in multi-waste valorization approaches, boosting ethanol titers obtained in biorefineries [113].

Biorefinery processes are used to reduce the negative environmental impact of agro-industrial waste by recovering valuable products through a combination of chemical, physical, and biological techniques. This process aims to achieve zero waste production of industries. In this context, a viable alternative for the valorization of whey is constituted by the integral use of whey within a biorefinery system. The whey biorefinery includes the recovery of lactoferrin, lactic acid, probiotics, hydrolyzed proteins and bioactive peptides, as well as the production of biogas from the remaining waste products [99].

Table 4 presents the various biorefinery systems used to valorize the cheese whey. Domingos et al. [114] indicated that integrated valorization of CW to produce high concentrations of polyhydroxyalkanoates is possible via the anaerobic fermentation of CW, and following an electrodialysis step. These generated biodegradable plastics can also be used to produce biopolymers. Sebastián-Nicolás et al. [99] proposed a three-stage biorefinery process including the separation of lactoferrin using a polyacrylamide matrix impregnated with copper, lactic fermentation using the residue resulting from the separation of lactoferrin, and the generation of biogas from the final residue.

Biorefineries that use agro-industrial waste are economically and environmentally sustainable. The biorefining of whey involves the use of physical, chemical or biotechnological processes to expand the product range of the dairy industry, while enabling the sustainable management of residual streams and reducing the disposal costs of final residues [10]. These systems combine the production of high-value products with the seamless integration of efficient waste treatment.

A whey biorefinery uses successive steps like filtration, fermentation, anaerobic digestion, etc., to recover proteins, lactose-based chemicals, and energy. This enables SMEs to generate revenue from a single side stream, thus avoiding the need to invest in costly final-stage treatments. Consequently, SMEs can target premium market segments such as functional foods, probiotics, bioactive peptides and green energy. These segments are becoming more popular with consumers and regulators due to their alignment with local branding and the concept of the circular economy [99,115].

SMEs can achieve profitability without the need for large-scale plants, as many biorefinery steps can be scaled to suit their throughput, e.g., membrane filtration, small-scale fermenters and micro-anaerobic digesters. Another advantage is that SMEs can reduce or replace external energy purchases by recovering energy. Furthermore, several SME cheese manufacturers have the option of joining forces by combining their whey in a shared biorefinery unit or cooperative facility. This approach reduces the individual capital and operating costs, while ensuring consistent whey quality for products that can be sold at a high price [12].

Whey biorefineries offer SMEs an attractive proposition, because a disposal problem is converted into a range of products and services using flexible, modular technologies that can be adapted to smaller volumes and shared infrastructure [115].

**Table 4:** Various biorefinery systems used to valorize the cheese whey.

Process Description	Substrate	Product	Process Condition/Efficiency	References
Two-stage biorefinery with anaerobic digestion	Whey and vinasse	Protein-rich fungal biomass and methane	Max. biomass yield: 12.0 g L^−1^ of wastewaters, cultivation at pH 6.5 and vinasse to whey ratio of 25:75 (*v*/*v*) for 96 h with nitrogen source supplementation.	[116]
Anaerobic fermentation of CW and electrodialysis (ED)	Cheese whey	Polyhydroxyalkanoates (PHAs)	PHAs yield: ca. 0.60 g PHAs g carboxylic acids^−1^ by fed-batch fermentation and 7 sequential batch ED processes.	[114]
Microbial fermentation by *Kluyveromyces marxianus* and *Saccharomyces fragilis*	Cheese whey	Ethanol and beta-galactosidase	Max. ethanol: 3.90% by *Saccharomyces fragilis* in 150 g L^−1^ CW at 40 °C, max. beta-galactosidase activity: 1.10 U mg^−1^ by *Kluyveromyces marxianus* in 150 g L^−1^ CW at 30 °C.	[74]
Two-stage bioprocess with microbial fermentation by *Saccharomyces cerevisiae* and *Gluconobacter oxydans*	Cheese whey powder (CWP)	Ethanol and galactonic acid	Max. ethanol: 110 g (1 kg CWP)^−1^, max. galactonic acid: 305 g (1 kg CWP)^−1^ for 24–36 h at 30 °C.	[75]
Ultrafiltration, reverse osmosis and fermentation	Tunisian cheese whey	Ethanol	Max. ethanol yield: 1.65 metric tonnes year^−1^, including a reverse osmosis procedure and fermentation at 28 °C for 20 h.	[117]
Centrifugation, filtration and fermentation by wild-type non-conventional yeast strains	Mizithra second cheese whey	Single-cell oil and polysaccharides	Total dry weight (TDW) of *Cryptococcus curvatus*: 22.0 g L^−1^, with 3.7 g L^−1^ cellular lipids produced (17% of TDW), 0.32 g L^−1^ h^−1^ biomass productivity, and 0.054 g L^−1^ h^−1^ lipid productivity in a 69 h long batch fermentation. TDW of *Papiliotrema laurentii*: 22.0 g L^−1^ and secreted significant quantities of exopolysaccharides.	[118]
Three-stage bioprocess, with dark fermentation, selection of PHA storing microorganisms, and PHA accumulation	Sheep cheese whey	Biohydrogen and PHA	Max. biohygrogen production: 5.3 L, and max. PHA production: 7.6 g per liter of sheep cheese whey at 39 °C and pH 6.0.	[18]

## 4. Global Whey Revolution: Examples of SME Innovations Worldwide

In Lebanon, companies such as Liban Lait and KMR Dairy have pioneered the valorization of whey through the UNIDO SwitchMed MED TEST III project by developing three products. One of these is a mint-flavored whey-based ayran (made with up to 50% acid whey and milk), which reduces production costs by 4–10%. The second product is a whey-based fruit juice (30% acid whey mixed with 70% apple juice or 40% lemon juice and 40% water), which achieves up to 25% lower production costs. The third product is a spreadable cheese made using sweet whey, which achieves 14–30% lower production costs compared to standard versions while enhancing nutrition [119].

In Europe, French SMEs such as Monts et Terroirs cheese dairy supply whey for innovative fermented drinks combined with fruit or vegetable juices, which were developed by INRAE-Rennes and Laboratoires Standa, produced by Enilbio in Poligny in collaboration with Sodiaal, with a zero-waste aspect using returnable bottles [120]. Spanish SME Central Quesera Montesinos converts surplus whey into biodegradable polyhydroxybutyrate bioplastics using a process of microbial fermentation via the WHEYPACK project, creating packaging for their own dairy products that replaces traditional plastics and lowers the environmental footprint [121]. In Italy’s Sardinia, small dairies process sheep milk whey from pecorino romano cheese via ultrafiltration to boost ricotta yield by 0.5%, alongside on-site protein concentrates for animal feed [122]. In Spain, the SME Formatgeria Montbrú in the Moianès region (Catalonia) valorizes goat whey waste from cheese production into high-value food supplements through an EU-funded CAP project. They have developed two innovative products—a whey-based dairy drink for athletes and a whey-based kefir—transforming otherwise discarded whey (with high COD of 70–80 g/L) into zero-waste by-products that enhance nutrition while cutting economic and environmental costs [123].

In addition, Ramos-Suárez et al. [64] has investigated the potential primary energy production from cheese whey in the Canary Islands. A total of 81.95 million liters of cheese whey are produced annually in the Canary Islands, which could be converted into 4.5 million m^3^ of biogas with the potential to contribute 108 TJ y^−1^ of renewable energy to the energy matrix. This study demonstrates that the valorization of cheese whey offers a promising alternative for small and medium-sized enterprises in the Canary Islands, providing an opportunity to replace fossil energy with biogas.

## 5. Conclusions

Most of the cheese whey in SMEs is discharged without any treatment, causing environmental issues, or is used as animal feed. However, the valorization of goat and sheep cheese whey in small dairy plants is important to provide the socio-economic and environmental aspects of sustainability.

The application of an integrated membrane process covering ultrafiltration/nanofiltration can be economically viable and feasible for small–medium-sized cheese plants to contribute to their sustainability.

According to the high biodegradability of cheese whey, biotechnological processes can be used as stand-alone systems or in combination with other physicochemical processes in SMEs, depending on the facilities of the dairy plant. Bioprocesses are important choices to valorize goat and sheep cheese whey produced by SMEs. Anaerobic digestion stands out for SMEs due to its proven economics and simplicity, while biorefineries offer greater potential but currently have lower commercialization potential. However, it is predicted that biorefinery systems will become more common in the future as they maximize outputs (biofuels, enzymes, biomass, PHAs, etc.). In addition, fermentation balances value and maturity for food-grade applications. Another innovative option is the production of functional beverages due to the increasing consumer demand for healthy foods.

## Figures and Tables

**Table 1:** Mean composition of bovine, ovine, goat whey and ovine second cheese whey (% *w*/*v*).

	Bovine CW	Ovine CW	Goat CW	Ovine SCW
Total solids	6.0–7.0	7.6–10.5	7.1–10.8	7.1–8.3
Lactose	4.2–5.0	4.3–6.1	5.0–6.7	4.5–5.7
Proteins	0.7–0.9	1.6–1.8	0.6–1.2	0.8–1.2
Fat	0.1–0.8	1.2–2.5	0.8	0.2–0.4
Minerals	0.5–0.6	1.0–1.8	0.6	1.7–1.9

CW: cheese whey; SCW: second cheese whey.

**Table 2:** High-value-added compounds generated from the cheese whey through fermentation over the last decade.

Value-Added Products	Growth Medium	Microorganisms	Process Condition/Efficiency	References
**Bioalcohols**				
Ethanol	Mashes containing 10% cheese whey powder	Eight yeast strains of *Lachancea thermotolerans*, *Kluyveromyces marxianus*, and *Kluyveromyces waltii*	*K. marxianus* achieved 3.5% (*v*/*v*) ethanol concentration at 96–120 h of fermentation. Also, mixed cultures and fed-batch fermentation technology increased efficiency.	[77]
Ethanol	Crude whey	*Kluyveromyces marxianus*	36 h at 30 °C, ethanol production: 10–12 g L^−1^ from crude whey, 17 g L^−1^ from 75% whey and 25% molasses.	[78]
Ethanol	Deproteinized cheese whey	*Kluyveromyces marxianus*	72 h at 20–35 °C, 35–100 g L^−1^ lactose concentration, and 180 rpm. Max. ethanol production: 35.5 g L^−1^ at 35 °C and 100 g L^−1^ lactose concentration.	[79]
Ethanol, 2-phenylethanol, isoamyl alcohol	Electro-activated whey/whey permeate	*Kluyveromyces marxianus*	96 h at 30 °C and 150 rpm, max. ethanol production: 28.13 g L^−1^.	[76]
**Bioprotein**				
SCP	Deproteinized cheese whey	*Kluyveromyces marxianus*	144 h at 20–35 °C, 35–100 g L^−1^ lactose concentration, and 180 rpm. max. biomass: 14.24 g L^−1^, SCP: 6.14 g L^−1^ at 20 °C and 70 g L^−1^ lactose concentration.	[79]
*Chlorella* biomass	Primary (PCW) and second (SCW) cheese whey from goat and sheep milk cheeses	*Chlorella* sp.	Chlorella cell growth was achieved in cheese whey with trace mineral addition, the measurements were carried out as chlorophyll-a content, max. value: 28.38 μg Ca (mL culture)^−1^ for undiluted PCW.	[80]
*Chlorella* biomass	Second cheese whey from goat and sheep milk cheeses	*Chlorella* sp.	Higher *Chlorella* biomass production (>800 mg mL^−1^) was achieved with trace mineral addition and continuous air supply.	[56]
Biomass	Electro-activated whey/whey permeate	*Kluyveromyces marxianus*	96 h at 30 °C and 150 rpm, max. biomass: 4.23 g L^−1^.	[76]
Biomass	Crude whey	*Kluyveromyces marxianus*	36 h at 30 °C and 3.8 pH, biomass: 6.20 g L^−1^.	[78]
Biomass	Cheese whey	Batch cultures *Geotrichum candidum*, *Penicillium corylophilum*, *Pleurotus ostreatus Penicillium restrictum*	3–6 days at 23 °C and 150–200 rpm, highest biomass yield: 16.9 dry weight L^−1^ by *P. corylophilum*.	[81]
**Biogas**				
Biohyrojen	Cheese whey	*Clostridium* sp.	24 h at 37 °C and 120 rpm max. biohydrogen yield: 6.35 ± 0.2 mol-H_2_/mol-lactose.	[82]
Biohyrojen	Cheese whey	*Lactobacillus* spp.	72 h at 37 °C and pH 6.5 with 18 h inoculum age, max. biogas: 85 mL 100 mL^−1^ of culture media.	[83]
**Organic Acid**				
Citric acid	Deproteinized cheese whey	*Kluyveromyces marxianus*	144 h at 20–35 °C, 35–100 g L^−1^ lactose concentration, and 180 rpm. max. citric acid: 8.3 g L^−1^ at 35 °C and 100 g L^−1^ lactose concentration.	[79]
Lactic acid	Renewable feedstocks: sugar cane bagasse, cheese whey, microalgal and macroalgal biomass	*Lactobacillus plantarum*	14 h at 30 °C with 200 rpm, 33.8 g L^−1^ lactic acid titer and 11.3 g (L h)^−1^ productivity.	[84]
Lactic, acetic, citric, propionic acids	Electro-activated whey/whey permeate	*Kluyveromyces marxianus*	Up to 96 h at 30 °C and 150 rpm, max. lactic acid: 1480.12 mg L^−1^, max. acetic acid: 1173.84 mg L^−1^, max. citric acid: 1908.43 mg L^−1^, max. propionic acid: 358.23 mg L^−1^.	[76]
Succinic acid	Whey and lactose	*Actinobacillus succinogenes*	72 h at 38 °C, 6.8 pH and 200 rpm, max. yield of 62.1% and max. productivity of 0.81 g L^−1^ h^−1^ were obtained with 35 g L^−1^ of whey.	[85]
Lactic, pyruvic, formic, acetic, butyric and propionic acid	Cheese whey	*Lactobacillus* spp.	24–64 h at 37 °C and 6.5 pH, max. lactic acid: 0.56 moles, max. acetic acid:0.12 moles, max. pyruvic acid: 0.031 moles, max. propionic acid: 0.165 moles, max. butyric acid: 0.01 moles, max. formic acid: 0.125 moles from the 0.114 moles of hexose.	[83]
Lactobionic acid	Ricotta cheese whey	*Pseudomonas taetrolens*	48 h at 30 °C and 150 rpm, max. lactobionic acid titer: 34.25 g L^−1^, max. yield 85% (mol lactobionic acid/mol of consumed lactose%).	[86]
**Enzymes**				
β-galactosidase	Cheese whey	*Saccharomyces fragilis*	72 h at 35 °C, 6.0 pH and 180 rpm with 20% inoculum concentration provided max. production of β-galactosidase.	[87]
**Aroma compounds**				
Diacetyl, Acetoin	Whey permeate	*Lactobacillus casei*	16 h at 37 °C, the production of acetoin (max: 12 mM) and diacetyl (max: 2.3 mM) was higher under respirative growth.	[88]
**Prebiotics**				
Galacto-oligosaccharide	Sweet whey	YO-MIX 101 culture (*Lactobacillus acidophilus*, *Streptococcus thermophilus* and *Bifidobacterium lactis*) and YO-MIX 532 culture (*Lactobacillus delbrueckii* subsp. *bulgaricus* and *S. thermophilus*)	24 h at 50 °C and 100 g L^−1^ initial lactose using YO-MIX 532 culture max. yield: 34.2% including disaccharide GOS, 25.4% excluding disaccharide GOS.	[89]
**Others**				
Carotenoids and chlorophyll	Ricotta cheese whey (bovine and ovine cheese whey)	*Chlorella protothecoides*	7 days at 25 °C, max. chlorophyll (42.17 mg L^−1^) and max. carotenoids (11.98 mg L^−1^) were found in the medium containing ovine ricotta cheese whey after 4 days of cultivation.	[90]

**Table 3:** Some studies on the development of whey-based beverages in the last decade.

Type of Beverage	Beverage Formulation	Reference
Fermented probiotic dairy beverage from goat whey powder	Reconstituted goat whey powder fermentation by co-culture of probiotic culture of *Lactobacillus casei* BGP93 co-cultured with *Streptococcus thermophilus* TA-40	[105]
Functional fermented whey-based beverage	Whey fermentation by lactic acid bacteria *Pediococcus pentosaceus* ENM104, *Lactiplantibacillus plantarum* SPS109 and their co-culture (1:1)	[106]
Whey-based pineapple mint beverage	Incorporation of *Mentha arvensis* extract into whey at different ratios (0 to 3%)	[102]
Whey-based mango herbal beverage	Incorporation of ginger extract (at different ratios between 0.5 and 5 ml (*v*/*v*)) and mango pulp into whey	[103]
Whey-based orange beverage	Incorporation of orange juice, sugar, stabilizer, citric acid and flavor into concentrated whey	[107]
Health based functional whey drink	Incorporation of carrot, beetroot and ginger juice, and mint extract into whey	[98]
Functional beverage	Mixing whey protein isolate (5%) with different concentrations of beetroot peel water extract (1, 2.5, and 5%) and flavored with strawberries puree (5%).	[108]
Functional fermented mulberry-whey beverage	Sweet whey fermentation by *Lactobacilllus bulgaricus*, *Streptococcus thermophilus* and probiotic cultures of *Lactobacilllus rhamnosus* GG and *Bifidobacterium animalis* ssp. *lactis*, and mixing with black mulberry juice at different ratios (0–100%)	[109]
Probiotic fermented whey-based beverages from cow, sheep and goat whey	Reconstituted cow, sheep and goat whey powder (separately) fermentation by *Streptococcus thermophilus* and probiotic cultures of *Lactobacillus acidophilus* and *Bifidobacterium animalis* ssp. *lactis*, and mixing with kiwi powder (1%)	[54]
Spirulina-fermented whey-based beverages	Fermentation of whey with *Streptococcus thermophilus*, and probiotic bacteria *Lactobacillus acidophilus*, and *Bifidobacterium* spp., fortified with Spirulina algae powder, and flavored with lemon and peppermint (*Mentha piperita*) juice	[110]
Passion fruit-flavored whey dairy beverages	Fermentation of whey and milk (70% milk/30% whey) with *Lactobacillus casei* and/or carbonation process flavored with passion fruit pulp	[111]

## Data Availability

No new data were created or analyzed in this study. Data sharing is not applicable to this article.
